# Insight into the intestinal microbiome of farrowing sows following the administration of garlic (*Allium sativum*) extract and probiotic bacteria cultures under farming conditions

**DOI:** 10.1186/s12917-020-02659-y

**Published:** 2020-11-13

**Authors:** Marta Satora, Marcin Magdziarz, Anna Rząsa, Krzysztof Rypuła, Katarzyna Płoneczka-Janeczko

**Affiliations:** 1 Department of Epizootiology with Clinic for Birds and Exotic Animals, Faculty of Veterinary Medicine, Wrocław University of Environmental and Life Sciences, Plac Grunwaldzki 45, Wrocław, Poland; 2 Hugo Steinhaus Center, Faculty of Pure and Applied Mathematics, Wrocław University of Science and Technology, Wyspianskiego 27, Wrocław, Poland; 3 Department of Immunology, Pathophysiology and Veterinary Preventive Medicine, Faculty of Veterinary Medicine, Wrocław University of Environmental and Life Sciences, Norwida 31, Wrocław, Poland

**Keywords:** Swine microbiome, Shaping, Garlic, *Lactobacillaceae*, Nutrition

## Abstract

**Background:**

Due to the tendency to reduce antibiotic use in humans and animals, more attention is paid to feed additives as their replacement. Crucial role of feed additives is to improve the health status, production efficiency and performance. In this original research, we estimate the potential influence of garlic (*Allium sativum*) extract and probiotic formula including *Enterococcus faecium*, *Lactobacillus rhamnosus* and *Lactobacillus fermentum* on the intestinal microbiota of sows, using the next generation sequencing method (NGS).

**Results:**

Our results indicate that the overall species richness as well as the composition of swine gut microbiota may be shaped by regular feeding with supplemented additives. On the Family and Genus level both additives (garlic extract and probiotics) seem to decrease microbiome diversity and richness. However, when it comes to garlic supplementation, we found the opposite trend on the Species level.

**Conclusions:**

The analysis of the selected microbial function indicates that both additives used in this study (garlic extract and composition of probiotics) seem to create a greater metabolic potential than estimated in a control group of sows. A general trend of losing or decreasing members of pathogenic species in the swine microbiome seems to occur in relation to both supplemented additives. In the prevention of some bacterial diseases supplemented additives could be considered for future use.

## Background

Over the last decade, the majority of scientists have been involved in research concerning the swine microbiome using differences in the bacterial 16S rRNA gene sequence and the next generation sequencing method (NGS). Isaacon and Kim presented a review of swine gastrointestinal (GI) microbiome, documenting a close relationship between the development of the microbiome and the sampling place within the GI tract as well as the growth of animals [1]. It has been confirmed in several studies that many agents have an influence on swine gut colonization by microorganisms starting from birth. They stem from the sows’ vagina, skin and faeces microbiome, through diet components, environment, infections and stress factors. Gastrointestinal microbiota constitute a dynamic structure which may evolve over time [2–4]. Holman et al. [5] prepared a meta-analysis report, based on previously collected data. Mentioned authors concluded that the results of the different studies on swine gut microbiota needed to be optimized, regardless of the country or place where the sample was taken (i.e. various parts of the gastrointestinal tract, where the samples were collected). The presence of yeast or viruses in the faecal samples of pigs was also studied using a culture-independent method [6, 7].

Taking into account health requirements and consumer protection, the development of antimicrobial resistance and the potential transfer of bacterial genes responsible for bacterial resistance between animals and humans as well as the possible environmental contamination, according to the implementation of EU legislation – antibiotics as growth promoters (AGPs) in animal feed have been banned from animal production in EU countries based on EC Regulation No. 1831/2003 [8, 9]. An issue of common concern among owners and veterinarians who are directly engaged in the economics of pig production as well as scientific researchers is therefore the possibility of implementing alternative substances that could have an influence on the efficient production of livestock. New feed additives have been developed, which are authorised in accordance with the same regulation (EC 1831/2003) as well as with Regulation No. 767/2009 of the European Parliament and of the Council regulates feed availability on the market and the use of feed, amending European Parliament and Council Regulation (EC) No. 1831/2003 and repealing Council Directive 79/373/EEC, Commission Directive 80/511/EEC, Council Directives 82/471/EEC, 83/228/EEC, 93/74/EEC, 93/113/EC and 96/25/EC and Commission Decision 2004/217/EC. With reference to the EC definition [10]: the role of feed additives used in animal nutrition is to improve the quality of feed, quality of food of animal origin or to improve an animal’s performance and health e.g. providing enhanced digestibility of feed components, correct its appearance, taste, smell or consistency. Therefore, the crucial role of the feed additives is to improve health status, production efficiency and performance. Among this group live probiotic bacteria cultures, prebiotics and synbiotics, acidifiers, feed enzymes, aromatic and flavouring agents, preservatives, antioxidants or yeast products could be found [11–14]. Their role is attributed to modulate or protect the immune system of pigs and participate in the regulation of the gut microbiota composition. Compounds isolated from plants also enjoy a great interest among feed additives (phytogenic feed additives, PFA) containing biologically active substances [15, 16]. One of them is garlic (*Allium sativum*) extract. The suitability of garlic-derived compounds has been evaluated in recent years in different species [17–22]. It has been shown that in broilers, the introduction of the garlic extract supplement has statistically significant potential to improve body weight gain in the first 4 weeks of life, compared with the control group supplemented with ciprofloxacin [20]. Based on the review paper by Senthilkumar et al. which includes numerous studies on the effectiveness of garlic extract, it could be concluded that garlic may enhance the feed conversion ratio (FCR) of these birds as well as decrease the mortality rate [21]. Supplementation of garlic may also influence the immunity of broilers, by increasing expression (mRNA) of the Toll-like receptors (TLR2, TLR4 and TLR7) and thus also make it possible to modulate the T-cell mediated immune response with garlic [23]. It has been confirmed that garlic or its derivatives can enhance the cellular and humoral immune system by stimulating some cells (e.g. macrophages, lymphocytes, NK) or mechanisms (e.g. immunoglobulin E production). It exhibits beneficial effects against microbial infections and anti-inflammatory activity [24] but its influence on production parameters is controversial. It was reported that garlic extract improves growth performance, nutrient digestibility and meat quality in pigs [25], however other authors did not observe such effects [26]. A garlic additive or its derivatives seem to be an interesting alternative to antibiotics. Tatara et al. confirmed that such additives facilitate good health status, performance and the systemic development of piglets [17].

Also, the use of probiotic bacteria as a feed additive for piglets and sows has been evaluated in recent years [27–29]. It is well documented that probiotics can reduce digestive disorders and improve productive parameters. They are especially recommended for piglets because of their rapid growth, immature immune system, digestive capacities and unstable intestinal microbiota. The largest group satisfying the definition of probiotics proposed by the International Scientific Association for probiotics and prebiotics (2013), are bacteria belonging to the Genera *Bacillus*, *Enterococcus*, *Lactobacillus*, *Pediococcus* and *Streptococcus*. In the European Union the QPC concept for probiotics (Qualified Presumption of Safety, including a history of safe usage and the absence of the risk of acquired resistance to antibiotics) has also been postulated [30]. The effects of probiotics in swine production has been evaluated in numerous publications. They boost the microbial composition in the gut and modulate the immune status. Moreover, they improve the average daily gain (ADG), average daily feed intake (ADFI) and feed conversion ratio (FCR), but there are still some uncertainties concerning their usage [31, 32]. These are mainly associated with the health status and maturity of various animals, farm conditions and kind of strains and proportions between them [11, 29, 33]. This dependency is not always identified and predictable which has prompted a search for new strategies and solutions. A bacterial strain of *Enterococcus faecium* was previously described in a study conducted by Napiorkówska et al. [34] where it was used in piglets and its impact on the composition of faecal microbiota was evaluated. The studies showed that the use of a probiotic compound in piglets had a positive impact on daily weight gain as well as a reduction of diarrhoea incidences. Piglets supplemented with *Enterococcus faecium* had an increased number of bacteria from the *Enterobacteriaceae* family, *Lactobacillus* spp., *Lactococcus* spp., and *S. cerevisiae* yeast were found. A reduction of diarrhoea incidences after the oral administration of a *Enterococcus faecium* supplement has also been observed by Zeyner and Boldt [35]. The adhesive capability of *Lactobacillus fermenti* 126 and *Lactobacillus rhamnosus* CCM 1825 has been evaluated by Brzozowski et al. [36]. It was concluded, that the analysed strains of lactic acid bacteria synthetize biosurfactant, whose molecules consist of proteins, polysaccharides and phosphates, which demonstrate good anti-adhesive properties against *Enterobacteriaceae*. *Lactobacillus rhamnosus* was also effective in ameliorating diarrhoea in weaned piglets [37]. Another study evaluated *Lactobacillus fermentum* effects on piglets’ growth, faecal microbiota, immune index and antioxidant activity [38]. Administration of the described probiotic bacteria increased the weight gain of piglets during the first 2 weeks of life, increased the number of *Lactobacillus* in faeces and increased the concentration of IgM, IgG, IgA as well as the activity of glutathione peroxidase in the plasma of the piglets.

Based on the literature review from the meta-analysis performed by Holman et al. [5] feed additives such as amylase and amylopectin, calcium phosphorus, distillers’ dried grains or resistant starch have been analysed and their influence on the gut microbiota has already been tested in the swine. The aim of this study was to investigate the potential influence of a garlic (*Allium sativum*) extract and probiotic formula including *Enterococcus faecium*, *Lactobacillus rhamnosus* and *Lactobacillus fermentum* on the intestinal microbiota of sows.

## Results

### DNA sequence data

A total of 5,738,676 DNA-identified sequences that were obtained in this study were then subsequently analysed in the metagenomic classification of the microbiota of farrowing sows, from the Kingdom to the Species taxonomic level. The DNA sequences were analysed for all individuals within groups, which was possible thanks to an individual bar code identifier for each pig sample. Table 1 illustrates the details regarding the number of identified sequences for individual sows and for the groups.

**Table 1:** Identified sequences for sows (A1-A8, B1-B8, C1-C8) and groups (A,B,C)

	Kingdom	Phylum	Family	Genus	Species
**A1**	27,246	26,609	24,920	24,455	13,648
**A2**	51,743	50,301	48,427	47,154	24,260
**A3**	49,218	48,271	46,663	45,712	27,148
**A4**	59,851	59,109	57,914	56,719	31,565
**A5**	54,671	53,934	52,103	50,945	30,475
**A6**	50,797	50,071	48,868	47,654	27,182
**A7**	62,604	61,989	60,309	58,953	30,083
**A8**	48,433	47,794	46,771	45,873	29,148
Group A					
**Total**	404,563	398,078	385,975	377,465	213,509
**Mean**	50,570.375	49,759.75	48,246.88	47,183.13	26,688.63
**SD**	10,687.7996	10,669.15	10,710.92	10,449.1	5758.181
**B1**	45,228	43,994	42,998	42,145	25,702
**B2**	108,414	106,150	103,524	101,310	52,313
**B3**	54,364	53,245	51,720	50,385	27,647
**B4**	59,115	57,891	56,009	54,638	28,518
**B5**	50,822	50,265	47,912	46,849	24,228
**B6**	57,400	56,846	53,653	52,548	27,342
**B7**	47,846	47,299	45,401	44,333	26,100
**B8**	46,936	46,299	43,581	41,531	22,325
Group B					
**Total**	470,125	461,989	444,798	433,739	234,175
**Mean**	58,765.625	57,748.63	55,599.75	54,217.38	29,271.88
**SD**	20,673.8936	20,175.28	19,936.02	19,617.94	9519.386
**C1**	57,695	56,221	53,479	52,223	28,243
**C2**	56,074	54,901	52,329	51,092	28,208
**C3**	61,661	60,692	59,245	57,684	27,546
**C4**	51,604	50,888	49,434	48,222	23,518
**C5**	61,213	60,729	55,946	54,754	31,255
**C6**	53,043	51,956	50,081	48,859	22,865
**C7**	49,755	48,949	47,359	46,107	23,856
**C8**	51,750	50,613	48,270	47,236	28,705
Group C					
**Total**	442,795	434,949	416,143	406,177	214,196
**Mean**	55,349.375	54,368.63	52,017.88	50,772.13	26,774.5
**SD**	4533.13953	4560.18	4072.079	3968.941	3000.356

Abbreviations: *SD* Standard deviation

### Microbial diversity

**Fig. 1:**
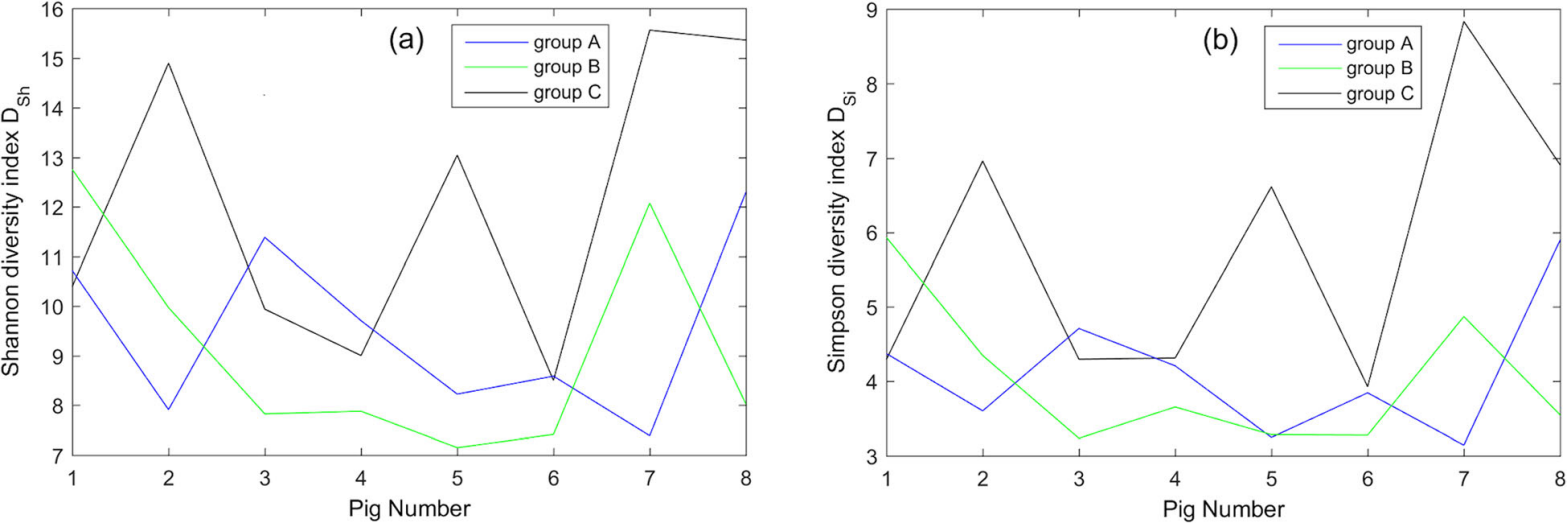
(**a**) Shannon and (**b**) Simpson diversity indices calculated on the level of Family taxonomic rank. For all eight pigs the corresponding Shannon and Simpson diversity indices on the level of Family rank were calculated and depicted. The comparison of the microbial diversity on the level of the Family taxonomic level shows the following trend – the most heterogeneous bacteria composition was estimated to be in control group C and the lowest diversities were observed in the group supplemented with probiotic bacteria (B). For the definitions of Shannon and Simpson indices see formulas (1) and (2) respectively

**Fig. 2:**
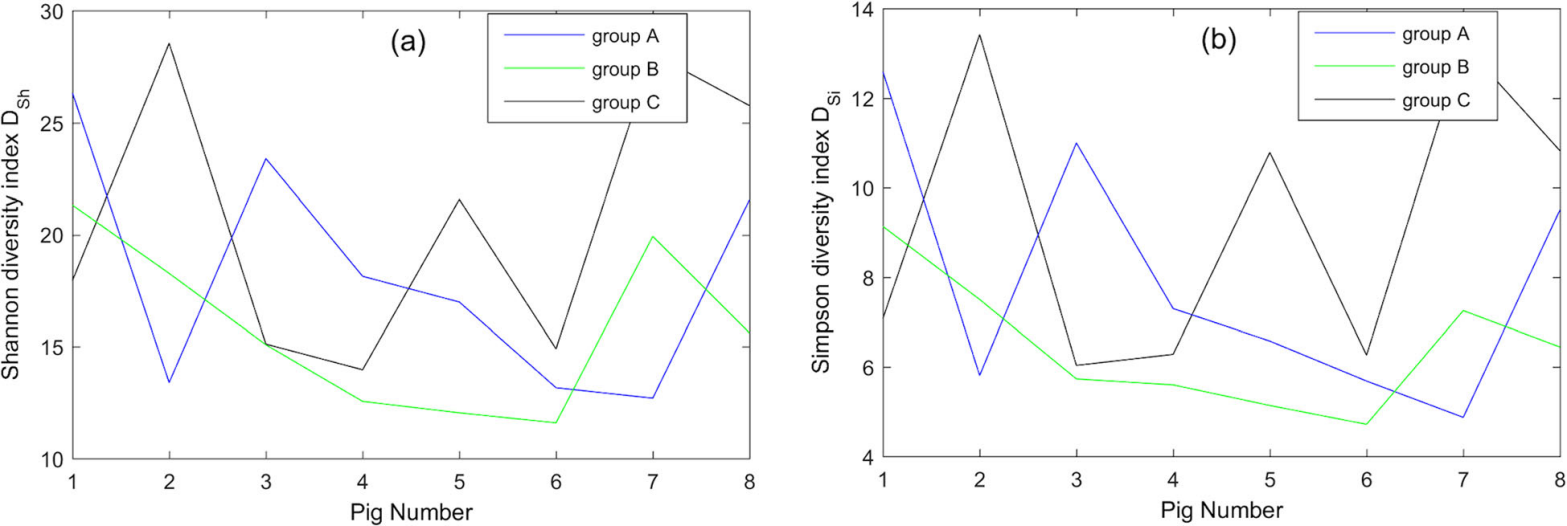
(**a**) Shannon and (**b**) Simpson diversity indices calculated on the level of Genus taxonomic rank. For all eight pigs the corresponding Shannon and Simpson diversity indices on the level of Genus rank were calculated and depicted. The comparison of the microbial diversity on the level of the Genus taxonomic level shows the following trend – the most heterogeneous bacteria composition was estimated to be in control group C and the lowest diversities were observed in the group supplemented with probiotic bacteria (B). For the definitions of Shannon and Simpson indices see formulas (1) and (2) respectively

**Fig. 3:**
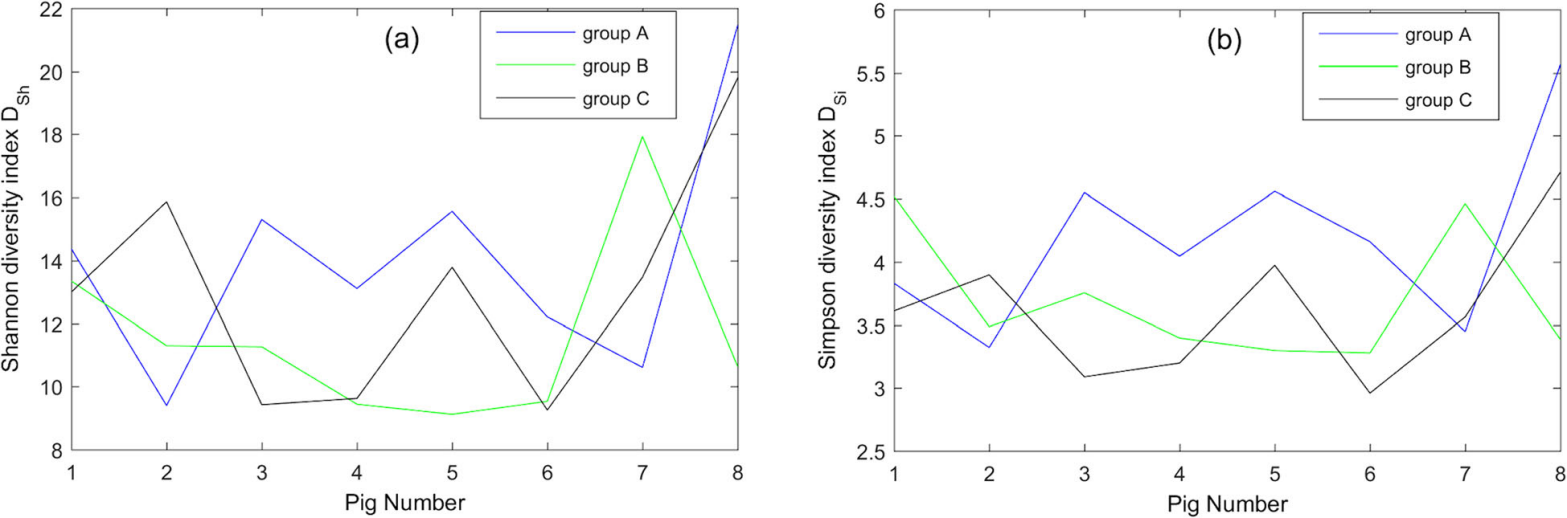
(**a**) Shannon and (**b**) Simpson diversity indices calculated on the level of Species taxonomic rank. For all eight pigs the corresponding Shannon and Simpson diversity indices on the level of Species rank were calculated and depicted. The tendency observed in Figs. 1 and 2 changed on the Species level – the highest microbial diversity was observed after the administration of the garlic extract (group A). For the definitions of Shannon and Simpson indices see formulas (1) and (2) respectively

The microbial diversity of farrowing sows after garlic or probiotic bacteria administration in all eight pigs in each group and the control group was measured using Shannon and Simpson diversity indices. The next Figs. 1, 2, 3 present the obtained results for each available taxonomic level (Family, Genus and Species).

In Table 2, details of the diversity measures (the average Shannon and Simpson diversity indices within groups) for the Family, Genus and Species the taxonomic levels are included.

**Table 2:** Comparison of microbial diversity between groups (A,B) in relation to the control group (C)

	Family		Genus		Species	
	Shannon index^a^	Simpson Index^a^	Shannon index	Simpson index	Shannon index	Simpson index
**Group A**	9.5278	4.1271	18.2079	7.9176	14.0041	4.1848
**Group B**	9.1371	4.0175	15.8000	6.4409	11.5741	3.6983
**Group C**	12.0881	5.7677	20.7367	9.2367	13.0290	3.6255

^a^ For the definitions of Shannon and Simpson indices see formulas (1) and (2) respectively

The comparison of microbial diversity takes into account the Family and Genus taxonomic levels highlighted in the same trend – the most heterogeneous bacteria composition was estimated to be in control group C and the lowest diversities were observed in the group supplemented with probiotic bacteria (B). However, this tendency changed on the Species level – the highest microbial diversity was observed after the administration of garlic extract (group A).

### Taxon-dependent analysis

A taxon-dependent analysis of the obtained results was performed to demonstrate possible shifts in the composition of faecal microbiota of sows administered with garlic extract and the composition of probiotic bacteria when compared to the control group. The results of the Kingdom distribution (Table 3) demonstrated that the proportion of *Bacteria* was almost identical in group A (92.64%) and C (92.89%) and slightly lower in the sows of group B that were supplemented with probiotics (91.03%).

**Table 3:** Relative abundance of microorganisms on the Kingdom level in sows group A, B and C

	Bacteria	Archeae	Unclassified	Other
Group A	92.4%	1.61%	4.29%	0.81%
Group B	91.03%	2.99%	4.93%	0.62%
Group C	92.89%	1.36%	4.94%	0.79%

**Fig. 4:**
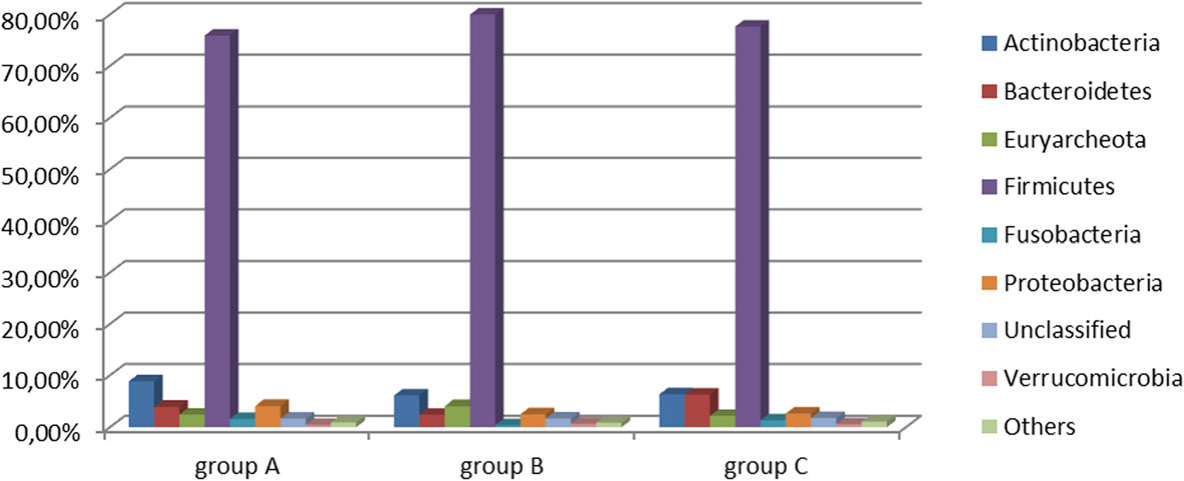
The mean relative abundance (%) of identified sequences on the Phylum level. The figure shows the detailed distribution of abundant microorganisms on the Phylum taxonomic rank. We observe that the proportion of Firmicutes in sows supplemented with garlic is slightly lower than in controlled pigs, however a significant increase in this group of microorganisms after probiotic supplementation.

A contrary tendency was visible, one that took into account the participation of *Archaea* – the largest proportion was in group B (2.99%) if compared with A (1.61%) or C (1.36%). For the needs of this study, at the subsequent taxonomic identification levels (Phylum, Family, Genus and Species) more than 1% of the total identified DNA sequences at least in one of the individuals was established as “abundant” as previously proposed by Kim et al. [39]. Several differences in the abundance of bacterial taxa between sows supplemented with garlic extract and a composition of probiotic bacteria were detected. Figure 4 shows the detailed distribution of abundant microorganisms on the Phylum taxonomic rank.

**Fig. 5:**
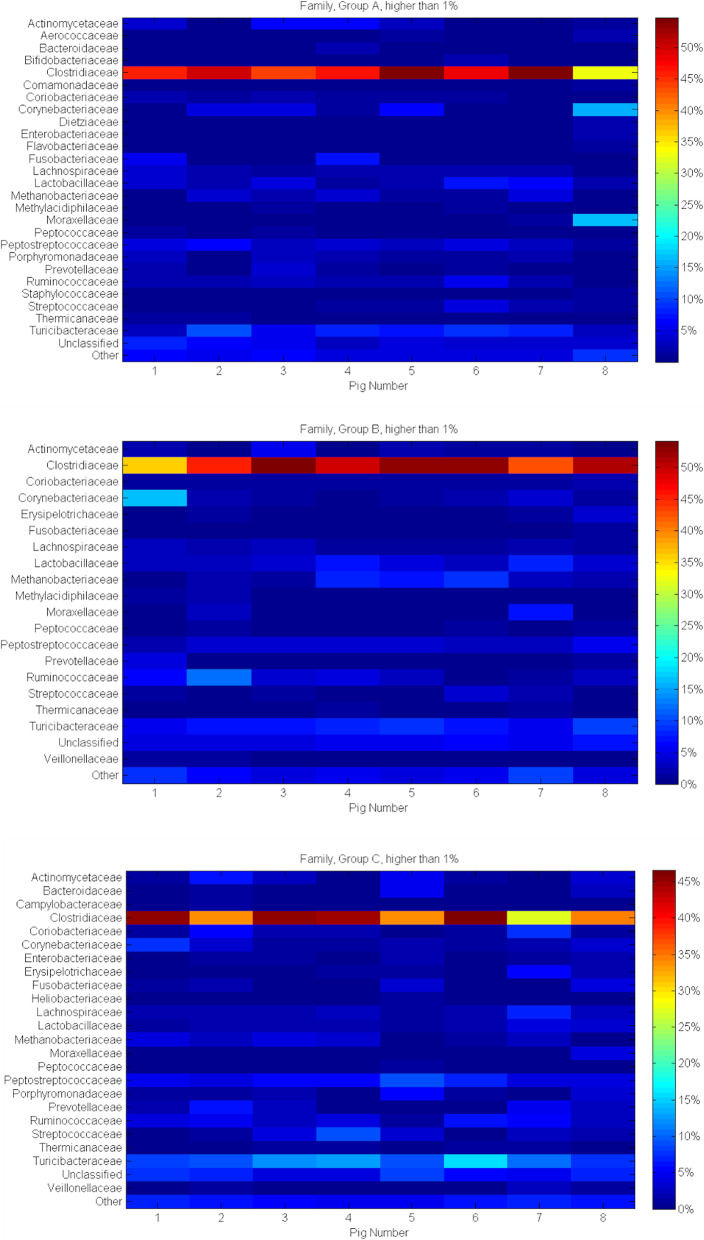
Prevalent Families analysis heat-maps of pig faecal microbiota considering feed additives and individual animals. The heat-map shows the relative abundance of identified sequences between individuals within the examined groups on the Family level. Density and saturation of each colour identify percentage (%) of sequences specific for analysed taxonomic rank. Presented are only sequences exceeding 1%. As ‘Other’ were marked the remaining sequences identified on the level lower than 1%

Two Families: *Clostridiaceae* (47.09, 48.04 and 39.02% in A, B, C respectively) and *Turicibacteriaceae* (6.84, 7.34 and 10.56% as previously) were mostly identified within all sow groups. The proportion of sequences that could not be assigned to the Family taxon was 4.89, 5.53 and 6.0% in the group A, B and C respectively. The other Families identified in proportion ≤ 1% in the same groups were 4.62, 4.09 and 4.58%. A total of 30 bacterial Families have been identified. Considering the differences, where at least in one of the sows within the Family taxon group could be identified as abundant (> 1%) 26, 19 and 23 Families were present for the A, B and C groups. For all three groups *Actinomycetacea, Clostridiaceae, Coriobacteriaceae, Corynebacteriaceae, Fusobacteriaceae, Lachnospiraceae, Lactobacillaceae, Methanobacteriaceae, Moraxellaceae, Peptococcaceae, Peptostreptococcaceae, Prevotellaceae, Ruminococcaceae, Streptococcaceae, Thermicanaceae* and *Thuricibacteriaceae* have been identified. In the sows supplemented with garlic extract in comparison with control groups, *Aerococcaceae*, *Bifidobacteriaceae*, *Comamonadaceae*, *Dietziaceae*, *Flavobacteriaceae*, *Methylabacteriaceae* and *Staphylococcaceae* have been shown. When comparing Families representative for sows supplemented with probiotic and control groups, the difference was recognised for the *Methylacidibacteriaceae* only, that were absent from the control group. Supplementation of garlic extract as well as probiotics may promote the absence of *Campylobacteraceae* and *Heliobacteraceae*. Garlic may also influence the absence of *Erysipelotrichaceae* and *Veilonellaceae*, that were recognised in the sows supplemented with probiotics and in the control groups. In sows fed with probiotics *Bacteroidaceae*, *Enterobacteriaceae* and *Porphyromonadaceae* have not been identified, although they were present in group A as well as C. Figure 5 shows detailed characteristics of microbiota within groups A, B and C on the level of Family rank.

*Clostridium* (31.89% in A, 35.65% in B and 28.75% in C) as well as *Turicibacter* (6.84% in A, 7.33% in B and 10.56% in C) were the most abundant Genera within all the groups. The proportion of sequences that could not be assigned to the Genus rank was 6.97, 7.94 and 8.25% in the groups A, B and C. The other Genera identified in proportion ≤ 1% in the same groups were 7.88, 7.21 and 7.80%. A total of 45 Genera have been identified in this study; 34 in group A, 23 in B and 37 in C respectively. For all three groups: *Acinetobacter, Actinomyces, Alcaliphilus, Anaerococcus, Arcanobacterium, Blautia, Clostridium, Corynebacterium, Fusobacterium, Lactobacillus, Methanobrevibacter, Mogibacterium, Oscillospira, Peptoniphilus, Prevotella, Psychrobacter, Ruminococcus, Sarcina, Streptococcus, Thermicanus* and *Turicibacter* were identified. In the sows supplemented with garlic extract in comparison with control groups, *Aerococcus*, *Bifidobacterium*, *Cetobacterium*, *Comamonas*, *Dietzia* and *Dysgonomonas* were detected. Garlic seems to support the presence of these Genera because they have also not been demonstrated in the sows supplemented with probiotics. However, when comparing Genera representative of control sows and the probiotic group, there was no characteristic Genus identified with regard to probiotics only. Both additives seem to promote *Coloramator* and *Candidatus Methylacidofilum*, Genera that were absent from the control group.

In sows that had been fed with garlic extract as well as with probiotics *Bulleidia, Campylobacter, Collinsella, Eubacterium, Faecalibacterium, Heliorestis, Mobiluncus, Natronincola, Olsenella, Oribacterium* and *Slackia* were not identified. Additionally, *Bacteroides*, *Escherichia, Peptococcus, Peptostreptococcus* and *Porphyromonas* were absent in sows from group B, although they were present in group A as well as C.

**Fig. 6:**
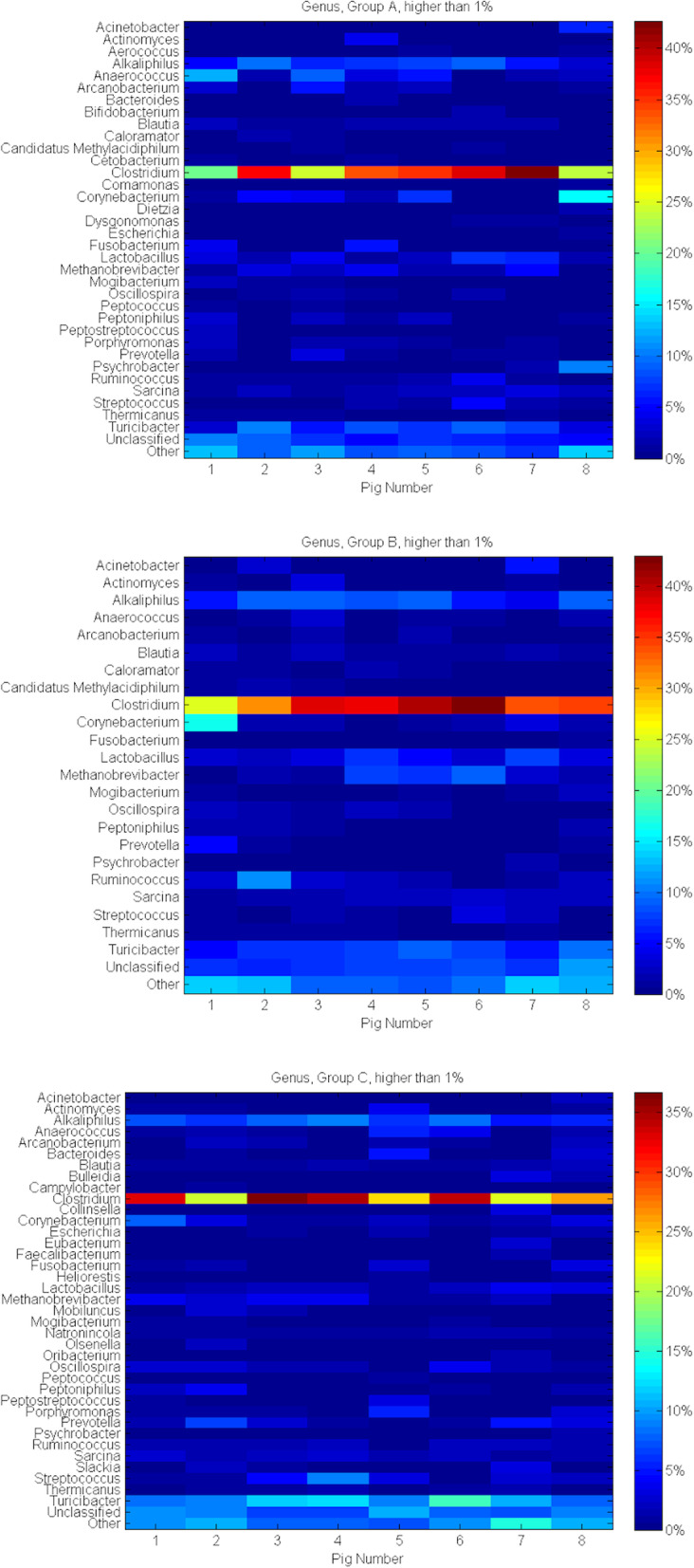
Prevalent Genera analysis heat-maps of pig faecal microbiota considering feed additives and individual animals. The heat-map shows the relative abundance of identified sequences between individuals within the examined groups on the Genus level. Density and saturation of each colour identify percentage (%) of sequences specific for analysed taxonomic rank. Presented are only sequences exceeding 1%. As ‘Other’ were marked the remaining sequences identified on the level lower than 1%

Figure 6 shows the detailed characteristics of microbiota within group A, B, C on the level of Genus rank.

*Clostridium cadaveris* (10.77% in A, 12.70% in B and 8.65% in C), *Alcaliphilus crotonatoxidans* (4.30% in A, 5.02% in B and 3.89% in C), *Clostridium butyricum* (2.21% in A, 2.15% in B and 1.63% in C), *Sarcina maxima* (2.18% in A, 2.11% in B and 1.81% in C) as well as *Lactobacillus ultunensis* (1.73% in A, 1.78% in B and 1.27% in C) and *Turicibacter sanguinis* (1.71% in A, 1.72% in B and 2.47% in C) were the most abundant Species within all three groups. The proportion of sequences that could not be assigned to the Species rank was 47.21, 49.81 and 51.62% in the group A, B and C. The other Genera identified in proportion ≤ 1% in the same groups were 14.26, 13.04 and 15.41%. A total of 43 Species have been identified in this study; 32 within group A, 19 in B and 29 in C, respectively. There were no differences between all three groups when it comes to the presence of *Acinetobacter lwofii, Actinomyces hyovaginalis, Alcaliphilus crotonatoxidans, Alcaliphilus peptidifermentans, Arcanobacterium pluranimalium, Clostridium butyricum, Clostridium cadaveris, Corynebacterium puruviciproducens, Corynebacterium xerosis, Lactobacillus ultunensis, Prevotella buccalis, Sarcina maxima, Streptococcus bovis* and *Thuricibacter sanguinis*. Garlic extract in comparison with control groups seems to support the presence of *Acinetobacter indicus, Aerococcus sanguinicola, Bacteroides sartorii, Bifidobacterium pseudolongum, Cetobacterium ceti, Clostridium saccharoperbutylacetonicum, Corynebacterium hansenii, Psychrobacter halophilus* and *Psychrobacter proteolyticus*, as these bacteria were also absent from the group fed with probiotics. When comparing Species representative for the probiotic group and control sows, probiotics may have an influence on the presence of *Lactobacillus antri* and *Lactobacillus reuteri,* which were absent as well from the sows supplemented with garlic extract.

Both additives seem to promote the occurrence of *Caloramator mitchellensis*, *Lactobacillus pontis* and *Ruminococcus bromii* that were undetected in group C.

**Fig. 7:**
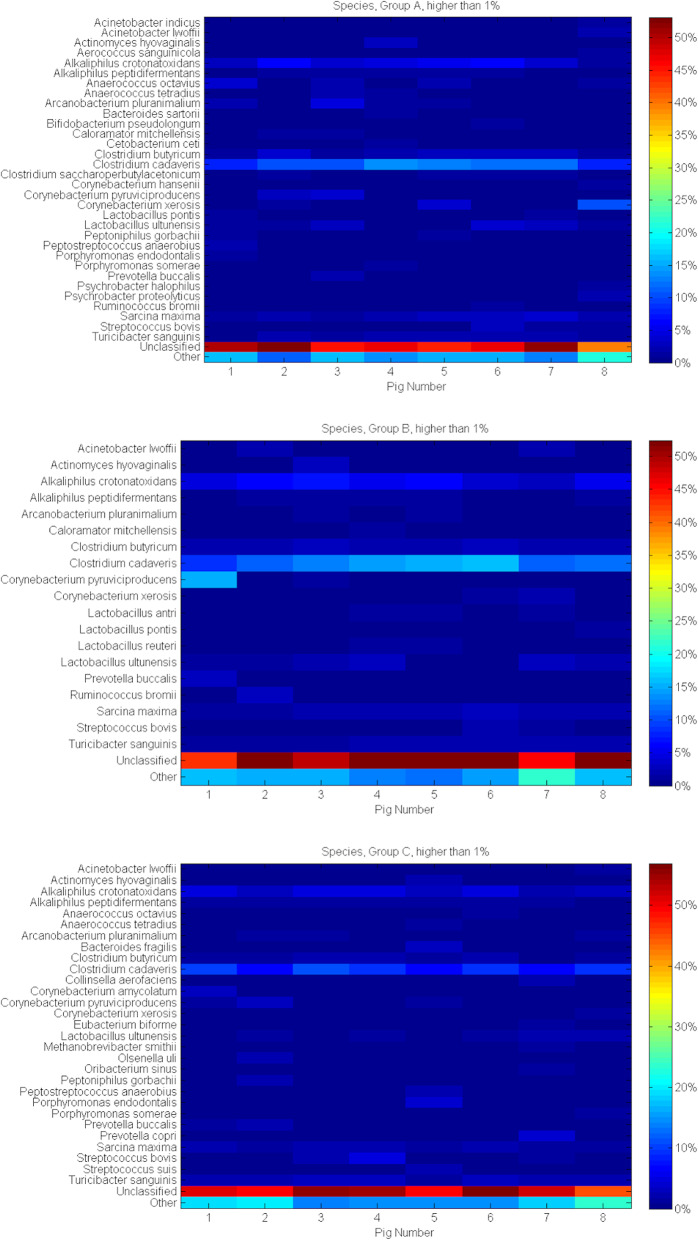
Prevalent Species analysis heat-maps of pig faecal microbiota considering feed additives and individual animals. The heat-map shows the relative abundance of identified sequences between individuals within the examined groups on the Species level. Density and saturation of each colour identify percentage (%) of sequences specific for analysed taxonomic rank. Presented are only sequences exceeding 1%. As ‘Other’ were marked the remaining sequences identified on the level lower than 1%

In sows fed with garlic extract as well as probiotics *Bacteroides fragilis, Colinsella aerofaciens, Corynebectarium amycolatum, Eubacterium biforme, Methanobrevibacter smithii, Olsenella uli, Oribacterium sinus, Prevotella copri,* and *Streptococcus suis* were not identified. Additionally, in sows fed with probiotics *Anaerococcus octavius* and *Anaerococcus tetradius*, *Peptoniphilus gorbachii, Peptostreptococcus anaerobius*, and *Porphyromonas endodonthalis* and *Porphyromonas somerae* were absent, although they were present in group A as well as in C. Figure 7 shows the detailed characteristics of microbiota within group A, B, C on the level of Species rank.

## Discussion

From the point of view of swine production (including both economic and health status of the animals) beneficial effects would have been apparent if modulation of the microbiome had led to the restriction of microorganisms, generally considered as pathogens or conditionally pathogenic. It would also be desirable to obtain such amendments in the microbiome. This would allow for better utilization of nutrients as well as a favourable energy balance that took into account metabolic processes.

Comparing our own results with those reported in other studies on pigs [40] the same tendency of high diversity in the microbial communities appeared. Differentiation in some taxa between experimental groups seem to have been connected with investigated additives which influenced the gastrointestinal microbiota; moreover, individual variability in the frame of the experimental groups was observed. It was also observed that the diversity and richness of the microbial community underwent a change together with the taxonomic level of identification. Some kinds of bacteria that were identified on the Family and Genus level, disappeared on the Species level. On the Family and Genus level both additives (garlic extract and probiotics) seem to decrease microbiome diversity and richness, however we found an opposite trend on the species level, taking into account garlic supplementation. Wide variations between presented taxonomic and functional shifts in the pigs’ gut microbiome is evident, based on the literature data. In the study by Homan et al. [5] among at least 90% of gastrointestinal samples, regardless of conditions, the genus *Clostridium, Blautia, Lactobacillus, Prevotella, Ruminococcus, Roseburia*, the RC9 gut group and *Subdoligranulum* were found. According to the authors cited above, these bacteria may serve as markers of a typical swine gut microbiota. Our results are in accordance with a large part of marked microorganisms, although Genus *Roseburia* or Genus *Subdoligranulum* have not been identified.

Quan et al. [41] compared gut microbiota of healthy fatteners which differed in daily gains, which resulted in a differentiation of FCR. Looking for potential relationships with FCR, functional profiles of gut microbiota seem to have appeared. Two digestion pathways, including carbohydrate metabolism (fermentation) and lipid metabolism were mainly represented in the colon. In the previous study on microbiome it was shown that the metabolism of *Archaea* is characterised by the absence of many classic routs, especially in carbohydrate metabolism – by which the degradation of some components differs strongly from typical pathways established by bacteria [42]. *Archaea* may also be involved in the methanogenesis (methane production by intestinal representatives) and different mechanisms of energy conservation, which could be beneficial in some conditions [43, 44]. Moreover, some studies have also demonstrated that *Archaea* seems to be a promising pharmabiotic, that can provide a supporting role even in the treatment of certain diseases, similarly to probiotics [45, 46]. Analysis of our data revealed that the supplementation of probiotics may have an effect on the increasing proportion of *Archaea* in the pig’s gastrointestinal microbiome, thus promoting the functional composition of the metagenome reshaped by diet additives. Bacteria that could be involved in metabolical pathways include *Prevotella* spp., *Clostridium*, *Blautia* and *Ruminococcus*, among others [5]. Bacteria in the genus *Ruminococcus* belong to important microbial symbionts, that have been found in numerous mammals, including pigs. The majority of well-known ruminococci utilise in their metabolism carbohydrates, including cellulitic and non-cellulitic specie**s** [47]. In our study, we found agreement between probiotic supplementation and the presence of *Ruminococcaceae* from the Family to the Species taxonomic level and identified species *Ruminococcus bromii* may facilitate the utilisation of resistant starches [48]. Recent studies have shown that an increased abundance of *Prevotella* may improve glucose metabolism, especially if the diet is enriched by fibre [49]. Changes in the abundance of *Prevotella* sp. were noted in pigs in response to diet, where during the nursing period and milk-oriented microbiome – there was only a small population of described taxa [50]. Exposure to garlic extract or probiotics in this study, did not enhance the prevalence of *Prevotella* sp. Additionally, the highest percentage of identification was observed in the control sows at every taxonomic level (*Prevotellaceae*, *Prevotella*, *Prevotella buccalis* and *copri*). Another microorganism that may provide energy from polisascharides that other gut microorganisms cannot decompose is *Blautia* (within the family *Lachnospiraceae*) [51]. In this study, garlic extract and probiotics did not show any modulatory effect on the prevalence of the genus *Blautia*. We also found that sequences characteristic of *Blautia* were not identified on the species level.

In recent years, attention has been paid to butyrate production with reference to its role in the maintenance of gut homeostasis and epithelial integrity and alternative ways of producing them need to be taken into account [52]. *Clostridiaceae* are an important group of bacteria for humans and animals, covering both commensal and pathogenic species. This family plays a significant role in the metabolic welfare of colonocytes, for which butyrate is an essential energy source. Alternative butyrate-producing pathways have been found in *Fusobacteria* and *Bacteroidetes* [52]. In our study, 59 Clostridium spp. were identified, whereas among abundant (> 1%) species, two were confirmed – *Clostridium butyricum* and *Clostridium cadaveris*. In both cases, the occurrence of *Clostridium butyricum* as well as *Clostridium cadaveris* increased with garlic supplementation as well as with probiotic additives, compared to the control group. The same tendency was visible on the genus level. *Clostridium butyricum* is human and animal common gut commensal bacterium, however in recent years particular attention has been paid to the role of this species – both beneficial and pathogenic [53]. Positive properties of this bacterium include its involvement in the production of high amounts of butyric acid, interleukin 1 and prevention of the development of acute colitis with the inflammation of the mucous membrane. However some strains of *C. butyricum* investigated in humans may be implicated in pathological conditions, including necrotizing changes [53]. *Clostridium cadaveris* is a commensal bacterium, selectively pathogenic in immunocompromised individuals [54]. Although several Clostridia are involved in the development of infectious diseases due to the production of toxins (*Clostridium perfringens*, *Clostridium difficle*), in the presented study, Clostridia considered to be pathogenic were identified as being not abundant (below 1% of the confirmed sequences for all groups). The metabolic activity of sows is also influenced by the occurrence in the methanogenic environment of *Alcaliphilus crotonatoxidans* that participate in the biochemical transformations of crotonates into butter and acetates [55]. In our study, *A.crotonatoxidans* was much more present in the supplemented groups than in control groups’ sows.

Another important and often analysed group of microorganisms are the *Lactobacillales*, a diverse and phylogenetically heterogeneous order of lactic acid-producing bacteria that include the genus *Lactobacillus,* composed of over 170 species. They utilise carbohydrate fermentation and produce lactic acid as a major end-product [56]. Several species and strains are considered to be among the most promising probiotics involved in the modulation of gut microbiota and prevention of pathological stages, especially thanks to their protective role against inflammatory intestinal diseases and reconstitution of barrier defects. A few studies also demonstrated an improvement in the gastrointestinal barrier function by the proliferation of harmful bacteria [57]. However, it is postulated that transient colonisation is often a typical attribute of lactobacilli and their survival in the GI tract is highly variable among strains. Based on the several human studies, faecal prevalence of Lactobacilli shows fluctuations and a lack of stability [58]. Our study did not reveal the presence of *Lactobacillus rhamnosus* and *Lactobacillus fermentum* as being administered to sows orally in group B, supplemented with those probiotic composition. There are possibly two reasons for this: the attachment to the mucosa of the preceding initial sections (jejunum, duodenum) where they may manifest their beneficial properties [59] or both strains may have a low stability of the presence taking into account faecal samples. Any conclusion is complicated by the fact that sows were sampled only once, during delivery. Despite this fact, supplementation of the proposed probiotic’s composition supports significantly the presence of the *Lactobacillaceae* in general taking into account the Family level and the Genus Lactobacillus compared with the control group of sows. Also, garlic extract improves participation of the above-mentioned microorganisms compared with the control group, although not as strongly as probiotics. As abundant inhabiting species in this study, we can consider *Lactobacillus ultunensis* only, because sequences characteristic for the remaining species (*L. antri, pontis, reuteri*) were not detected above 1% in all groups. Of the remaining species *L. reuteri* is widely described in the literature, as bacteria able to produce a variety of antimicrobial substances such as lactic acid, hydrogen peroxide, reuterin and reutericyclin, which exhibits beneficial effects for the organism. In the pig industry, the administration of *L. reuteri* resulted in an improvement in the growth rate, feed efficiency and reduction of diarrhoea incidence in neonatal and growing pigs [60].

In our study we also observed some tendencies taking into account non-abundant taxa (< 1%) of metabolic bacteria like *Acinetobacter indicus, Bacteroides sartorii, Bifidobacterium pseudolongum, Cetobacterium ceti, Clostridium saccharoperbutylacetonicum, Corynebacterium hansenii, Psychrobacter halophilus* and *Psychrobacter proteolyticus*. The majority of these bacterial species involved in metabolic changes in the intestinal lumen and supplementation of garlic extract seems to promote their participation in the microbiome composition. Bifidobacteria (among them *Bifidobacterium pseudolongum*) are generally considered to promote intestinal health by preventing the colonisation of potential pathogens: they reduce intestinal pH through an increased amount of fermentation products, produce inhibitory substances like bacteriocins and stimulate the immune system [61].

Analysis of the selected microbial function based on the literature data indicate that both additives used in this study (garlic extract and composition of probiotics) seems to create a greater metabolic potential than estimated in the control group of sows. Similarly, to other authors [39, 50, 62] that confirmed some shifts in the gut microbiome after the supplementation of antibiotics, we estimated a similar tendency for supplemented probiotics as well as garlic extract. Importantly, proposed additives generate a selection of specific bacterial populations that contribute to the metabolism of animals. Further studies are needed to explain if estimated shifts in the composition of metabolic bacteria may also have a direct influence on the improvement of a pig’s growth or fattening performance. It is also essential to understand whether or not the detected shapes of the microbiome after the addition of garlic or probiotic supplementation affect the cross-feeding relationship between individual members of the microbiome.

Another important aspect of our study was to find out if the supplementation of garlic extract, or a composition of probiotic bacteria, could modulate gut microbiome in such a way as to influence the typical pathogenic bacteria. Soler et al. [63] adopted colistin sulphate and amoxicillin to estimate the effect of supplemented microbials on the digestive microbiota. They analysed the influence of antibiotics starting with the higher taxonomic ranks of microbiome such as Phylum and have demonstrated a relative increase in *Bacteroidetes* in conjunction with a decrease in *Firmicutes*. In our study, phytobiotic properties of garlic extract as well as probiotics used in pigs seem to be oriented towards a decrease of *Bacteroidetes.* The proportion of *Firmicutes* in sows supplemented with garlic is slightly lower than in controlled pigs but we noted a significant increase in this group of microorganisms after probiotic supplementation. This does not, however, change the fact that in our study Gram-positive *Firmicutes* as well as Gram-negative *Bacteroidetes* represent a dominated Phila, described as being essential sign of healthy adult microbiota. Relation and proportion between this two Phyla (*Bacteroidetes*: *Firmicutes*) may contribute to disease states, however high variability in this proportion has been observed [64]. The relevance of the described ratio is therefore debatable in humans. In veterinary medicine, a lower *Bacteroidetes*: *Firmicutes* ratio was established for diarrheal piglets [65] but we did not observe any clinical disorders in the examined sows.

Administration of tested antimicrobials in those research studies seems to have an influence on *Lactobacillus* spp. depletion with the reduction of aerobic and anaerobic bacteria, especially Gram-negative bacteria. The researchers determined a decrease in the abundance of *Proteobacteria* and *Lactobacillus* in the antibiotic-treated group and a shift in the abundance of the Family *Prevotellaceae* species, which cover the emerged niche [63]. In our study, garlic extract as well as the probiotics have the opposite impact on *Lactobacillaceae*. We observed an increase in the abundance of this Family in both of the supplemented groups. We also confirmed an opposite shift for the Family *Prevotellaceae*. Administration of both supplemented additives may have an effect on the *Prevotellaceae* reduction. In the analysis of the sequences identified within Family and Genus taxon rank, garlic extract as well as probiotics seem to reduce participation of Streptococci. On the Species level *Streptococcus suis* (etiological agent of streptococcosis, that can be isolated from the lungs, brain, heart and joints of infected animals) was confirmed in the controlled sows (group C) only and was not identified in pigs supplemented with garlic or probiotics. *Streptococcus suis* is one of the most important pathogens in pigs causing arthritis, meningitis, septicaemia and many other infections, what is more it is a crucial zoonotic agent responsible for septicaemia, meningitis and other infections in humans [66]. So, in the prevention of streptococcal disease supplemented additives could be considered for future use. *Actinomyces hyovaginalis*, bacteria that may be associated in pigs with disseminated necrotic lung lesions, was identified as being non-abundant in all groups [67]. Out of the bacteria that may have an influence on an animal’s health status, single papers describe *Bacteroides fragilis, Colinsella aerofaciens, Corynebacterium amycolatum* or *Olsenella uli.* Garlic extract as well as probiotics had an influence on the participation of *Bacteroides fragilis*, that was not identified in all animals from groups A and B. *B. fragilis* is considered to be an opportunistic pathogen, however it may exhibit both pathogenic and beneficial properties in the host [68]. In pigs, enterotoxigenic strains were isolated from the intestinal contents of animals with diarrhoea [69]. Additionally, both additives show the possibility of reduction in *Colinsella aerofaciens* occurrence. This bacteria can be linked to health disorders such as irritable bowel syndrome in humans but we did not find any information that would link its participation to similar disorders in pigs [70]. *Corynebacterium* constitute a part of the normal flora of animals while others are opportunistic bacteria. Described by Wendt et al. [71] *Corynebacterium suis*, associated with urinary disease in pigs, were not identified in our study. In this study, differences between groups were evident in the richness of the selected species like *Corynebecterium amycolatum (*absent from groups A and B) and *C*.*hanseni* (absent from group B) – microorganisms that could be isolated from wounds and pus respectively [72, 73]. It is also noticeable that *Corynobacterium xerosis*, described as isolated mostly from subcutaneous lesions in swine, occurred in our study most frequently in sows fed with garlic extract [74]. In turn, in association with probiotics we noted a higher prevalence of *Corynebacterium puruviciproducens*, bacteria which exhibits immunomodulatory properties and can stimulate hosts humoral immune response to pathogenic microorganisms by adjusting the function of macrophage [75]*. Olsenella uli* was isolated from an inflamed human mouth and occasionally from the blood of humans with local oral or gastrointestinal infections [76] and in our own study bacteria participated in the microbiome of control sows only. From a clinical perspective *Campylobacteraceae*, particularly species like *Campylobacter coli*, *C. jejuni* or *C. lari* may have an epidemiological significance in pigs (diarrhoeal disease) as well as in humans [77]. In our study, *Campylobacteraceae* were detected on just the Family and Genus taxonomic rank and only covered controlled sows. However, we did not find sequences characteristic for pathogenic species in the following steps of taxonomic identification. The lowest frequency of *Erysipelotrichaceae* was confirmed in sows supplemented with garlic extract, however the disappearance of Genus *Erysipelothrix* was observed in all groups. We also did not identify typical swine pathogenic species *Erysipelothrix rusiopathiae*.

## Conclusion

To conclude, a general trend of losing or decreasing members of pathogenic species in the swine microbiome seems to occur in relation to both supplemented additives. Our study strongly supports the hypothesis that the intestinal microbiome of sows may be heavily and favourably shaped by the supplementation of garlic extract and examined probiotic composition. Probiotics seem to boost both the presence of *Archaea* and the participation of Firmicutes. As a result of probiotics supplementation, the highest biodiversity was noted within detectable *Lactobacillus* species. Probiotics seem to influence also the highest proportion of *Corynebacterium puruviciproducens* (known for its immunomodulatory properties) and *Ruminococcus* spp. (identified as important microbial symbionts). Garlic extract appears to create the general highest biodiversity of the sows’ microbiome on the Species level. Supplementation of garlic may also limit the presence of pathogenic bacteria, taking into account *Erysipelotrichaceae*.

Further research upon feed additives is still needed because diet ingredients as well as microbe-microbe interactions are factors modulating gut microbiome composition. Therefore, the domination of some species in parallel with the disappearance of others may constitute a risk of dysbiosis also in pig production.

## Methods

The trial was performed on a private commercial pig farm, where the sow herd consisted of 130 animals. Farm owner has consented to the study. The feed for the sows was individually dosed using an electronic sow feeding system (Wolbrink NEDAP, Netherlands). The standard feed used for sows on the farm was as follows:

Sows before mating up to a week before farrowing (1 ton): 50% barley, 22.5% triticale, 20% rye, 5% soybeans, 2.5% premix* Polfamix LP TOP1^a^ (Trouw Nutrition Polska Ltd.)

*a Polfamix LP TOP1: Lysine 4 g, Methionine 2 g, Threonine 2 g, Total Calcium 297 g, Total Phosphate 69 g, Sodium 62 g, Magnesium 10 g, Vitamin A 480000 IE, Vitamin D3 65,000 IE, Vitamin E 3000 mg, Vitamin K 80 mg, Vitamin B1 80 mg, Vitamin B2 200 mg, Vitamin B6 150 mg, Vitamin B12 1200 mcg, Biotin 8000 mcg, Niacin 1250 mg, Folic Acid 200 mg, Ca Pantothenate 600 mg, Choline 12,000 mg, Manganese 3100 mg, Zinc 5000 mg, Iron 4000 mg, Copper 990 mg, Iodine 30 mg, Selenium 12 mg, Betaine 5500 mg, Antioxidant 1000 mg.

Sows a week before delivery up to about a week after weaning piglets (1 ton): 50% barley, 30% wheat, 15% soybeans, premix* 4% Polfamix LK TOP2^b^ (Trouw Nutrition Polska Ltd.) 0.7% soybean oil, 0.3% feed acidizer, JHJ Baracid (JHJ Sp. o.o.). In addition, after weaning – until next mating sows receive 5% glucose to feed (200 g / animal / day).

*b Polfamix LK TOP2: Lysine 50 g, methionine 7 g, Threonine 8 g, Total calcium 230 g, Total Phosphorus 60 g, Sodium 55 g, Magnesium 10 g, Vitamin A 300000 IE, Vitamin D3 50,000 IE, Vitamin E 3000 mg, Vitamin K 100 mg, Vitamin B1 55 mg, Vitamin B2 225 mg, Vitamin B6 100 mg, Vitamin B12 1000 mcg, Biotin 10,000 mcg, Niacin 700 mg, Folic Acid 50 mg, Ca Pantothenate 500 mg, Choline 12,000 mg, Manganese 2500 mg, Zinc 2500 mg, Iron 3000 mg, Copper 625 mg, Iodine 30 mg, Selenium 12 mg, Betaine 5500 mg.

On day 80 of the pregnancy twenty-four pregnant crossbred sows (Polish Landrase x Polish Large White) were randomly selected and assigned to one of three groups: A (*n* = 8) – supplemented with garlic (*Allium sativum*) extract; B (n = 8) – supplemented with probiotic bacteria; and C (n = 8) – the control group, without any additional supplementation. Only the negative control was included in the study. The positive control (sows supplemented with antibiotics) was not included in this research due to the EU-wide ban on the use of antibiotics as antimicrobial growth promoters (AGP) in animal feed (entered on January 1, 2006).

In group A the garlic extract additive (Allivet™, solution of natural garlic oil extract, level of analytical constituents: crude fat – 0.71%, sodium – 24 mg/kg; Centaur, Poland) was administered in a dose of 10 ml/sow every 3 days, from the 80th day of gestation to the weaning day, according to the manufacturer’s recommendation.

Probiotic bacteria *Enterococcus faecium*, *Lactobacillus rhamnosus* and *Lactobacillus fermentum* were administered in a dose of 2 g/sow/day from the 80th day of gestation to the weaning day. The strains of probiotic bacteria *Enterococcus faecium* CCM 6226 (serial number: 030714), *Lactobacillus rhamnosus* CCM 1825 (serial number: 010914) and *Lactobacillus fermentum* CCM 7192 (serial number: 011014) were selected from a collection of microorganisms of PharmaGal-Bio (Slovakia) – all strains are admitted to trading as feed additives (EC Regulation 1831/2003) and are on the updated list from 24.10.2018 [78]. The mixture of probiotic bacteria was prepared integrating corn starch (690 g), maltodextrin (50 g) and protein (250 g) with 10 g of the probiotic bacteria (1 × 10^9^ CFU/g) in every 1 kg. Both additives were fed individually by direct oral administration.

Due to the fact that the preparations are approved for use in animals, after the study sows remained for breeding use on the farm.

On the day of delivery (during farrowing), faecal swabs were collected for DNA extraction and sequencing analysis using sterile rectal swabs (Deltalab, Poland). DNA extraction and all procedures related to the new generation sequencing (NGS) analysis were performed in GENOMED S.A. (Warsaw, Poland). Genomic DNA from each sample was isolated using Genomic Mini AX Bacteria (A&A Biotechnology, Poland) according to the manufacturer’s instruction, with an additional mechanical lyses of each sample supported by zircon balls in a FastPrep® homogenizer (MP Biomedicals, Poland). Each swab was placed in a tube containing 500 μl of the BS buffer for bacteria suspension, adding 20 μl of lysozyme and 5 μl of mutanolysin (lysozyme and mutanolysin provide a synergistic action by increasing the effectiveness of lysis of the bacterial cells from the Genera *Streptococcus*, *Lactobacillus*, *Lactococcus*, *Listeria*)*.* Then samples were mixed and incubated in the Eppendorf TermoMixer® (50 °C, 20 min, 1400 RPM (revolutions per minute). The suspension (400 μl) was transferred to centrifuge tubes and shaken with zirconia balls (60s, BeadBeater©, Biospec Products). Finally, 1 ml of lysis buffer (LS) and 20 μl of proteinase K were added.

Bacterial DNA was confirmed using real-time PCR in the thermocycler Stratagen Mx3000P (Thermofisher Scientific, USA) and SYBR Green (Sigma Aldrich, USA) as a fluorochrome. For the amplification of the fragment of bacterial gene 16S rRNA, the universal primers have been used [79]. The PCR-mixture and the time-temperature profile were as below (Table 4).

**Table 4:** Composition of the PCR mixture and temperature profile for the bacterial gene 16S rRNA amplification

Real Time 2x-PCR Mix SYBR A	10 μl
Starter 1055F 10 μM 5′-ATGGCTGTCGTCAGCT-3’	0.4 μl
Starter 1392R 10 μM 5′-ACGGGCGGTGTGTAC-3’	0.4 μl
DNA template	1 μl
deionized water	8.2 μl
initial denaturation	95 °C, 3 min.
denaturation	95 °C, 15 s
priming	58 °C, 30 s
elongation	72 °C, 30s
appointment of the melting curve (measurement of the fluorescence in each temperature)	65 °C - > 95 °C

Abbreviations: *PCR* Polymerase chain reaction, PCR Mix SYBR A – ready-to-use mixture to the real-time PCR with fluorophore (A&A Biotechnology, Poland); F = forward primer; R = reverse primer

Hypervariable regions V3-V4 of the 16S rRNA gene were amplified. For amplification of the selected region and the preparation of the DNA libraries a pair of primers 341F and 785R were used. The PCR reaction was performed using NEBNext® Q5 Hotsart High-Fidelity DNA Polymerase (NEB). Table 5 shows the time-temperature profile for the DNA libraries.

**Table 5:** The time-temperature profile for metagenomic analysis of the 16S rRNA V3-V4 hypervariable region

NGS PCR profile			
Step	Temperature	Time	No. of cycles
initial denaturation	98 °C	30 s	1
denaturation	98 °C	10 s	3–15
annealing	65 °C	75 s	
final extension	65 °C	5 min	1
final hold	4–10 °C	∞	

Abbreviations: *16S rRNA* 16S ribosomal RNA, *NGS* Next generation sequencing, *16S V3-V4* Regions of interest-specific primer

The new generation sequencing was performed in a MiSeq Reagent Kit v2 (Illumina) sequencer and the data analysis performed using MiSeq Reporter (MSR) v2.6 software and the 16S Metagenomics protocol. The analysis consisted of three parts: the automatic demultiplexing of samples, the generation of fastq files containing raw readings and classification of reads (paired-end type) in the various taxonomic categories. The 16S Metagenomics protocol ensures species-specific classification of the obtained readings based on the reference sequences database Greengenes v13_5, modified by Illumina. Preparation of the reference database involved: filtering of the sequences shorter than 1250 bp, a filtering of the sequences containing more than 50 degenerated bases (M, R, W, S, Y, K, V, H, D, B, N); filtering the not fully classified sequences (the lack of classification on the taxonomic “genus” or “species” level). Taking into account that possible shifts in the intestinal microbiota after garlic or probiotic bacteria administration have been examined, data analysis obtained from the NGS was performed, including protocols with all sequences after filtering.

The diversity of microbial communities within groups A, B, C and between groups using sequence reads was analysed using two standard diversity measures: Shannon and Simpson diversity indices [80].

For a given community the Shannon diversity index is defined as

1 $$ \kern1em {D}_{Sh}=\exp \left(-\sum \limits_{i=1}^R{p}_i\ln {p}_i\right) $$

Here *R* is the richness of the community (i.e. the total number of types in the community), whereas *p*_*i*_ is the proportional abundance of the *i*-th type. *D*_*Sh*_ is the exponent of the Shannon entropy and it measures the level of uncertainty in the community. *D*_*Sh*_ ranges from 0 (when all the individuals are of the same type) to *R* (when all *p*_*i*_ = 1/*R*).

The Simpson diversity index is defined as:

2 $$ {D}_{Si}=\frac{1}{\sum \limits_{i=1}^R{p_i}^2}\kern2.75em $$

It measures the degree of concentration of the community (more precisely, it is the reciprocal of the probability that two individuals taken at random from the community are of the same type). Both diversity indices were calculated for all eight pigs from each group A, B and C. The calculations were done separately on each available taxonomic level (Family, Genus and Species). All the calculations were performed in the MATLAB R2016a environment. The obtained results allowed us to compare the microbial diversity between the analysed groups.

## Data Availability

Raw data for calculation of tables and figures are available from the corresponding author upon request.

## Abbreviations

ADFI: Average daily feed intake
ADG: Average daily gain
AGP: Antibiotic growth promoter
bp: Base pair
BS buffer: Bacterial suspension buffer
DNA: Deoxyribonucleic acid
EC: European Commission
EU: European Union
FCR: Feed conversion ratio
GI tract: Gastrointestinal tract
IgA: Immunoglobulin A
IgG: Immunoglobulin G
IgM: Immunoglobulin M
LS buffer: Lysis buffer
mRNA: Messenger ribonucleic acid
MSR: MiSeq Reporter
NEB: NEBNext® Q5 Hotsart High-Fidelity DNA Polymerase
NGS: Next generation sequencing
NK cells: Natural killer cells
PCR: Polymerase chain reaction
PFA: Phytogenic feed additive
QPC: Qualified Presumption of Safety
rRNA: Ribosomal ribonucleic acid
RPM: Revolutions per minute
SD: Standard deviation
TLR: Toll-like receptors
